# Endocrine and Metabolic Pathways Linked to Keratoconus: Implications for the Role of Hormones in the Stromal Microenvironment

**DOI:** 10.1038/srep25534

**Published:** 2016-05-09

**Authors:** Tina B McKay, Jesper Hjortdal, Henrik Sejersen, John M Asara, Jennifer Wu, Dimitrios Karamichos

**Affiliations:** 1 Department of Cell Biology/ University of Oklahoma Health Sciences Center, Oklahoma City, OK 73104, USA; 2 Department of Ophthalmology, Aarhus University Hospital, Aarhus C DK-8000, Denmark; 3 Division of Signal Transduction, Beth Israel Deaconess Medical and Department of Medicine, Harvard Medical School, Boston, MA USA; 4 Department of Ophthalmology/Dean McGee Eye Institute, Oklahoma City, OK 73104, USA

## Abstract

Hormones play a critical role in regulating tissue function by promoting cell survival, proliferation, and differentiation. Our study explores the influence of endocrine function in regulating metabolism and inflammatory pathways in Keratoconus (KC), which is a corneal thinning disease associated with reduced stromal deposition. KC is known to be a multifactorial disease with an elusive pathogenesis. We utilized a cross-sectional study analyzing clinical features and saliva samples from sixty-four KC patients and fourteen healthy controls. In order to determine if endocrine function varied between healthy controls and KC, we measured hormone levels in saliva and found significantly increased dehydroepiandrosterone sulfate (DHEA-S) and reduced estrone levels in KC patients compared to healthy controls. We measured significant variations in metabolites associated with pro-inflammatory processes, including myoinositol and 1-methyl-histidine, by targeted mass spectrometry. We also measured significantly increased IL-16 and stem cell factor in KC saliva samples compared to healthy controls, with higher expression of these pro-inflammatory proteins correlating with increased KC clinical grade, corneal curvature, and stromal thinning. Our results identify a novel mechanism linking KC and pro-inflammatory markers and suggest that altered hormone levels modulate metabolism, cytokine, and growth factor expression leading to increased severity of the KC condition.

Hormone production occurs in the major endocrine and exocrine organ systems in the human body, including the pituitary gland, thymus, pineal gland, thyroid, pancreas, ovaries, testes, adrenal glands, salivary glands, and sweat glands^1^. Endocrine secretions are released directly into the bloodstream where they circulate to organs and tissues via the lymphatic and blood vasculature. Hormones have been reported to play a significant role in various pathologies, including autoimmune disorders^2^,^3^ and cancer^4^. The gender-dependence of many of these diseases is correlated with variations in sex hormones, such as estrogen and testosterone^5^. These steroid-based hormones are synthesized in the smooth endoplasmic reticulum or mitochondria from precursors that are secreted systemically. Dehydroepiandrosterone sulfate (DHEA-S) is a common precursor to other androgens including androstenediol, androstenedione, testosterone, and dihydrotestosterone, which are generated following the conversion of DHEA-S to dehydroepiandrosterone (DHEA) in a tissue-dependent manner^6^. Studies have shown that DHEA-S levels do not vary with gender but are significantly reduced with aging^7^. Supplementation with DHEA-S has been studied clinically to determine its effects in improving cognitive function, depression, and fatigue^8^,^9^. Furthermore, DHEA-S has been shown to modulate patient outcome in inflammatory diseases, such as Lupus^10^ and Sjögren’s syndrome^11^, with females composing 88% and 94% of the patient population, respectively^12^. Hormones are also known to have profound effects on regulating lacrimal gland function and dry eye development^13^. Androgen and estrogen receptors have been identified in the human cornea^14^ and retina^15^ suggesting that hormones regulate tissue function within the anterior and posterior segments of the eye as well.

The most common corneal dystrophy in the US is Keratoconus (KC), which is a corneal thinning disease that affects over 1 in 2000 people worldwide and results in corneal ectasia and significantly impaired vision^16^,^17^. Though not considered an overt inflammatory disorder due to the lack of clinical signs, KC has been associated with upregulated pro-inflammatory cytokines, interleukin-6 (IL-6), tumor necrosis factor-α (TNF-α), and matrix metalloprotease-9 (MMP-9), isolated in tears from KC patients^18^,^19^. Furthermore, eye rubbing due to ocular irritation is the most common etiological feature occurring in over two-thirds of KC patients^20^,^21^. These studies suggest that an inflammatory response may play an important role in the progression of KC leading to thinning of the stroma.

Normal corneal stromal structure is assembled and maintained at an average thickness of roughly 532–610 nm varying from person to person^22^,^23^. In the KC cornea, the central corneal thickness is reduced 8–30% with averages reported of 447.8 μm (±57.8)^24^. Even slight thinning of the corneal stroma can lead to a bulging phenomenon that distorts transmission of light to the retina. In KC the eye becomes cone-like at the region with increased thinning near the corneal apex in the presence of normal intraocular pressure that is maintained by fluid flux. Variations in hormone levels have been shown to have direct effects on corneal thickness in healthy individuals^25^. Reports have been published as to the gender differences within the KC population^26^,^27^,^28^. However, to the authors’ knowledge, investigation into alterations in hormone levels in KC has not been explored. The purpose of our study was to elucidate the role of hormones and inflammation in the KC pathology by addressing the following questions: Is there a correlation between KC severity and hormone levels?, 2) Is systemic metabolism altered in KC that may drive a pro-inflammatory response?, and 3) Which cytokines or growth factors are elevated in KC leading to a more severe clinical presentation? In order to do so, we utilized a non-invasive method that has never been used before for the diagnosis or treatment of KC. Saliva screening together with clinical observations was used here to determine its correlation to KC.

The long-term goal of these studies is to identify biomarkers that contribute or drive KC pathogenesis in order to assist in early diagnosis and enable development of therapeutics to treat this visually debilitating disease. Previous studies have identified inflammatory markers in KC^18^,^19^, however, to the authors’ knowledge, no studies have investigated systemic inflammation and KC severity. Based on the early age of onset^29^ and gender dominance^26^,^27^,^28^ of KC, we hypothesized that altered hormone levels may play a role in KC development, with inflammatory factors contributing to a more severe disease phenotype. This impactful work explores how systemic changes in hormone levels can contribute to stromal deposition defects seen in KC.

## Results

### KC Patient Demographics

**Figure 1:**
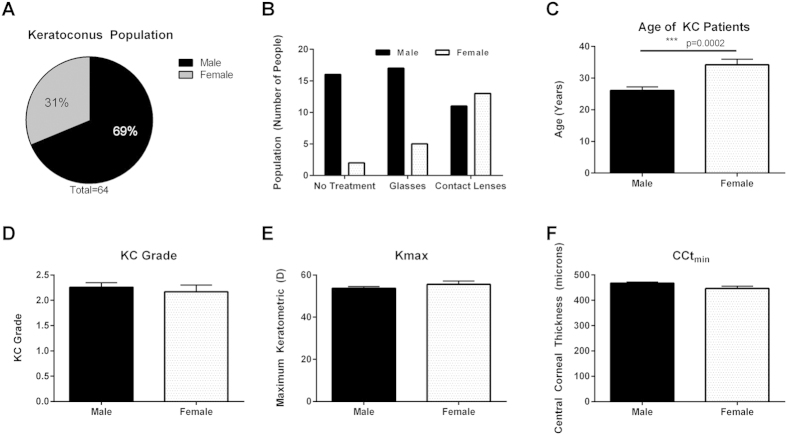
Demographics of the Keratoconus (KC) population based on gender. (**A**) In a total of 64 patients, 44 (69%) were identified as male with 20 (31%) were identified as female suggesting increased male dominance in the KC population. (**B**) Distribution of KC patients based on treatment for correction for visual acuity deficits due to KC in male KC patients. (**C**) Average age of KC patients divided by gender. Gender correlations with (**D**) severity of KC as clinically diagnosed as KC grade, (**E**) measure of central corneal curvature (Kmax), and (**F**) minimum central corneal thickness (CCt_min_). Statistical significance was measured using a Mann-Whitney two-tailed T-test with p ≤ 0.05 considered statistically significant. Error bars represent standard error of the mean. n = 64 total patients, with the clinical parameters for each KC eye tabulated in (**D–F**), total number of eyes ≥ 110.

In a total of 64 KC patients, 44 (69%) were identified as male and 20 (31%) were identified as female (Fig. 1A). Since KC leads to significant defects in visual acuity, most patients are prescribed glasses or contact lenses to correct vision^30^. We classified the distribution of KC patients based on treatment for correction for visual acuity deficits due to KC in both genders (Fig. 1B). The distribution of subgroups based on prescribed treatment modality within the KC male population including, no treatment (36%), glasses (39%), and contact lenses (25%) did not vary between treatments (Fig. 1B). However, within KC female patients, contact lens wearers (65%) were considerably higher compared to the glasses-prescribed subgroup (25%) or no treatment (10%) suggesting a correlation between gender and favored treatment modality (Fig. 1B). The KC population also showed a significant increase in average age (>8 years) in the females with KC at 34 ± 1.75 years old compared to their male counterparts (26 ± 1.14 years) (Fig. 1C, p = 0.0002). The KC population in our study was male-dominated with increased age of female KC patients. A significant reduction in central corneal thickness (CCt_min_) and increased maximum corneal curvature (Kmax) are indicative of KC diagnosis and are often proportional to KC severity or grade. In order to determine if either gender correlated with differential severity of disease, we analyzed KC grade, CCt_min_, and Kmax based on gender (Fig. 1D–F). No statistical difference was observed between gender and severity suggesting that though KC is male-dominated, gender does not directly modulate KC severity (Fig. 1D–F). Further analysis of clinical features (KC grade, Kmax, CCt_min_) and treatment for visual acuity (no treatment, glasses, contact lenses) showed no statistical variations between treatment modality and severity of KC (Supplemental Fig. S1A,B). However, a subpopulation of the contact lens wearers had substantially higher corneal curvature (Kmax) values compared to the mean of the population (Supplemental Fig. S1C). Contact lenses may contribute to KC progression by promoting local hypoxia within the central cornea^31^.

### Altered hormone levels in KC

**Figure 2:**
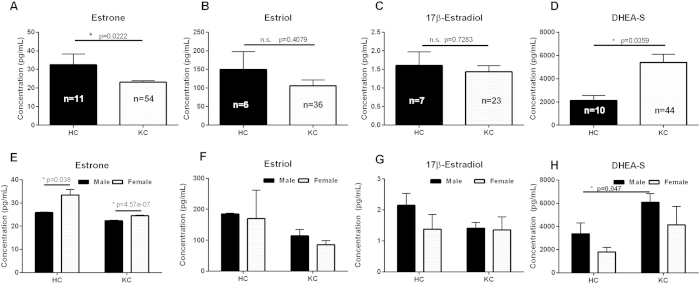
Salivary hormone levels measured in healthy controls (HC) and Keratoconus (KC) patients. (**A**) Estrone, (**B**) estriol, (**C**) 17β-estradiol, and (**D**) dehydroepiandrosterone-sulfate (DHEA-S) levels measured by enzyme-linked immunosorbent assay (ELISA). A significant reduction in estrone levels were measured in KC with increased DHEA-S levels and no variations in estriol and 17β-estradiol levels between HC and KC. (**E–H**) Breakdown of hormone levels based on gender. Statistical significance was determined using a Mann-Whitney two-tailed T-test with p ≤ 0.05 considered statistically significant. Error bars represent standard error of the mean. n ≥ 6 for all samples, with each n representing a different patient. All patient samples were analyzed for each hormone present, and only samples with concentrations within the range of the standard curve were plotted.

Systemic changes in estrogen and androgen levels have been shown to directly modulate corneal thickness during pregnancy and development^25^,^32^,^33^. Surprisingly, to-date, no studies have reported their link in KC. In order to determine the role of hormones in KC we quantified concentrations of estrone, estriol, 17β-estradiol, and DHEA-S found in saliva samples (Fig. 2). Our results showed a significant reduction in estrone (1.3-fold, p = 0.0222) levels in KC saliva samples compared to healthy controls (Fig. 2A) with no significant differences in estriol or 17β-estradiol levels (Fig. 2B,C). DHEA-S levels were significantly higher (2.5-fold, p = 0.0359) in KC samples compared to controls (Fig. 2D). Analysis of hormone levels based on gender was used in order to determine the differences within HC and KC groups. As expected, we found a female-dependent elevation of estrone in both the healthy controls and KC patients compared to their male counterparts (Fig. 2E, 1.6-fold and 1-fold, respectively, p ≤ 0.038). Furthermore, estrone levels were reduced in KC female patients compared to the healthy female controls. The estrogen-derived hormones, estriol and 17β-estradiol, were not dependent on gender in our population and slight reduction was seen in KC patient samples from both genders compared to controls (Fig. 2F,G). Our results show that both male and female KC patients had increased DHEA-S levels (1.8-fold, p = 0.047) compared to the healthy controls supporting a role for elevated DHEA-S and reduced estrone in KC pathogenesis (Fig. 2H). Our data shows that there were significant variations in estrone and DHEA-S in the female and male KC population, respectively, which attribute to altered endocrine activity and KC. In order to determine if the systemic concentration of one hormone modulated that of another, we plotted ratios of hormone levels for KC patients compared to healthy controls. We measured a robust linear response between estrone and estriol levels in both groups (Supplemental Fig. S2), even though the mean estrone levels in KC patients were significantly lower than healthy controls (Fig. 2). Moreover, the increased DHEA-S levels did not correlate with increased estrone, estriol, or 17β-estradiol suggesting that systemic levels of estrogens are independent of DHEA-S production, and therefore increased DHEA-S levels may have a tissue-dependent response within the cornea that may contribute to the KC pathology.

### Hormone Levels and KC Severity

**Figure 3:**
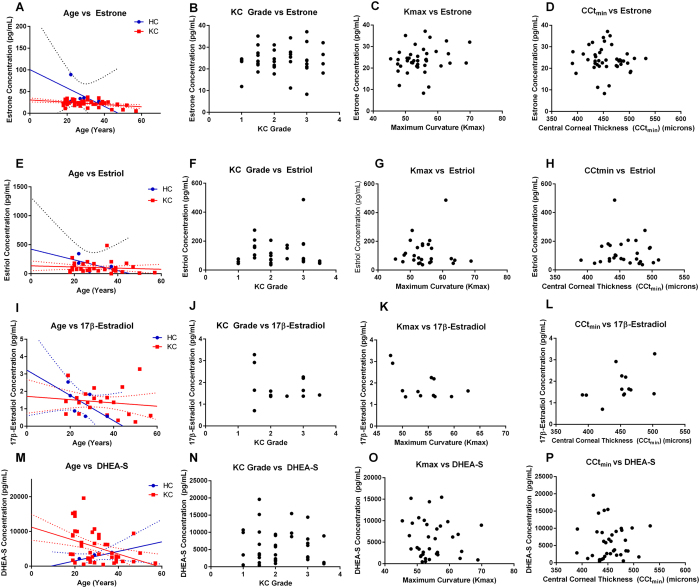
Hormone levels relative to clinical presentation characterized by KC grade, central corneal thickness (CCt_min_), maximum curvature (Kmax), and in Keratoconus (KC) patients compared to healthy controls (HC). (**A–D**) Estrone, (**E–H**) estriol, (**I–L**) 17β-estradiol, and (**M–P**) DHEA-S levels plotted relative to age at time of collection, clinical severity (KC grade), maximum curvature (Kmax), and minimum central corneal thickness (CCt_min_) measured in microns. For each patient, the eye with the most severe KC clinical features was plotted versus hormone concentration. (**A,E,I,M**) Best-fit linear curve for age-dependence of hormone concentrations shown with solid lines and the 90% confidence interval with dotted lines (HC in blue and KC in red).

In order to determine if varying degrees of severity of KC can be correlated with altered hormone levels, we analyzed estrone, estriol, 17β-estradiol, and DHEA-S levels relative to age, KC grade, maximum curvature (Kmax), and minimum central corneal thickness (CCt_min_) (Fig. 3). Both estrone and estriol levels were linear with a slight reduction with increases in age in both healthy controls and KC patients (Fig. 3A,E). Neither KC grade, Kmax, or CCt_min_ correlated with estrone or estriol levels (Fig. 3B–D, F–H). 17β-estradiol levels were more variable with age in KC patients with slightly lower levels in KC grades greater than 2, high Kmax (>50), and thin central corneal thickness (<425 microns) (Fig. 3I–L). DHEA-S levels also gave a linear reduction with age in KC patients with no correlation with KC grade, Kmax, or central corneal thickness (Fig. 3M–P). These results suggest that a complex interplay exists between hormonal levels and severity of KC with altered endocrine function modulating other factors, such as metabolism or inflammation.

### Role of Metabolism in KC

**Figure 4:**
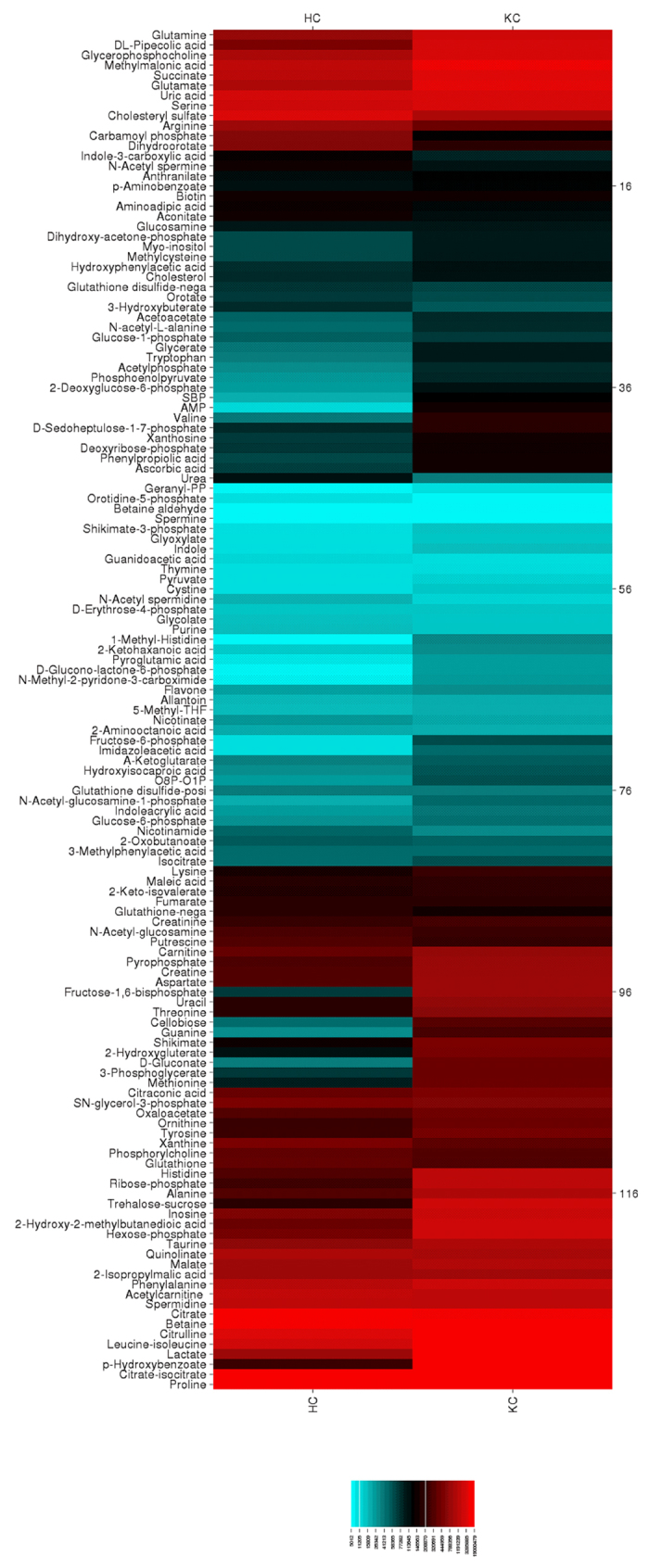
Heat map of metabolites isolated in saliva from healthy controls (HC) and Keratoconus (KC) patients isolated by LC-MS/MS. Metabolites present at high concentrations are depicted in red with moderate concentrations shown in black, and low metabolites in blue. n = 4 for each group, with each n representing a different patient.

**Figure 5:**
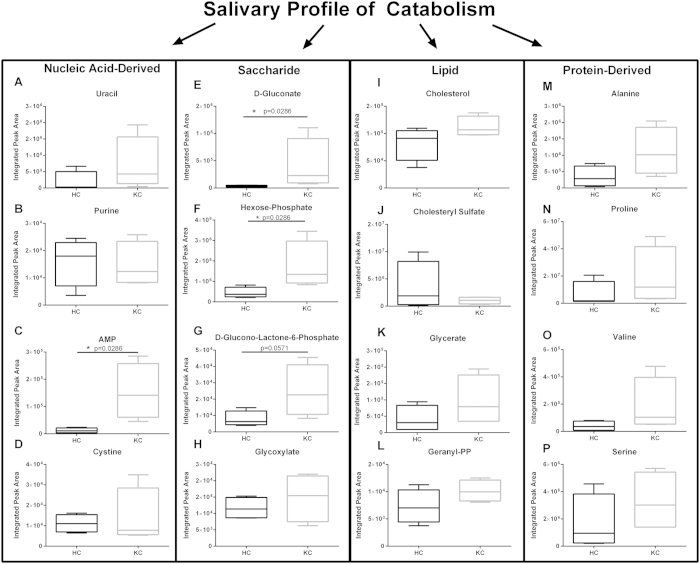
Salivary profile of the major classes of biomolecules measured in healthy controls (HC) and Keratoconus (KC) patients. (**A–D**) Nucleic acid-derived, (**E–H**) saccharide-derived, (**I–L**) lipid-derived, and (**M–P**) protein-derived metabolites were measured by LC/MS-MS. Significantly elevated AMP and D-glucono-lactone-6-phosphate were measured in KC saliva samples compared to HCs. n = 4, with each n representing a different patient. The boxplot representation depicts the central rectangle spanning the interquartile range with a line showing the median. Whiskers above and below the central box represent the maximum and minimum data points, respectively. Statistical analysis was performed using a Mann-Whitney two-tailed T-test with p ≤ 0.05 considered statistically significant.

KC has been reported to have significant differences in cellular metabolism within corneal stromal cells that may give rise to increased oxidative stress and bioenergetics^34^,^35^. We have previously identified a significant reduction in adenosine triphosphate (ATP) production via oxidative phosphorylation and an increase in aerobic glycolysis favoring lactate production in primary cells isolated from KC patients^35^. Elevated lactate levels are associated with hypoxic conditions with a lowering of the extracellular pH and increased oxidative stress and apoptosis^36^, which are both associated with KC^34^,^35^,^37^,^38^. In order to determine if systemic changes in metabolism contribute to KC development, we quantified the concentrations of metabolites (Supplemental Fig. S4) found within saliva using shot-gun tandem mass spectrometry^39^ and found significant variations in metabolite flux suggesting a potential link to altered systemic metabolism and KC. As shown in the heat map (Fig. 4), the most dominant metabolites present within human saliva from both healthy and KC individuals were uric acid, citrate, betaine, and proline (Fig. 4). Furthermore, the greatest variations in metabolite flux between KC and healthy controls were linked to glucose metabolism. In order to determine if altered glucose-derived metabolite levels led to variations in metabolic flux in other pathways, we determined the salivary profile of metabolites derived from the four major classes of biomolecules: nucleic acid, saccharide, lipid, and protein (Fig. 5). We measured significant variations in adenosine monophosphate (AMP) (12.7-fold), D-gluconate (12.3-fold), and hexose-phosphate (3.9-fold) in KC saliva samples suggesting variations in nucleic acid and glucose metabolism (Fig. 5C,E,F, p = 0.0286). Lipid-derived metabolites, including cholesterol, cholesteryl sulfate, glycerate, and geranyl-pyrophosphate (PP), did not vary between healthy controls and KC samples (Fig. 5I–L). Moreover, protein metabolism was also not altered in the disease samples suggesting that hormonal differences in KC primarily modulate glucose metabolism independent of lipid- or protein-breakdown.

Since our results suggested that glucose metabolism was altered in the KC pathology, we investigated the conversion of glucose to lactate or pyruvate in order to determine if oxidative phosphorylation was modulated (Supplemental Fig. S3A–C). We found substantial increases in lactate:malate (5-fold, p = 0.0951) and lactate:pyruvate (4-fold, p = 0.0659) in KC saliva samples suggesting that glucose is converted to the anaerobic glycolytic product, lactate, rather than proceeding through the more efficient mode to generate adenosine triphosphate (ATP) via the citric acid cycle and oxidative phosphorylation. Altered cellular metabolism can contribute to increased intracellular oxidative stress with glutathione as the primary antioxidant responsible for reducing reactive oxygen species. Since KC has been correlated with increased oxidative stress within the cornea^34^,^35^,^40^ and *in vitro* by our group^35^, we measured salivary concentrations of reduced (GSH) and oxidized glutathione (GSSG) levels in order to determine if systemic GSH levels are affected in KC (Supplemental Fig. 3D,E). We measured a significant reduction in GSH (p = 0.0384) and a slight reduction in GSH:GSSG (p = 0.1055) in KC saliva samples compared to healthy controls suggesting systemic elevated oxidative stress may be important in KC (Supplemental Fig. 3D–F).

### Systemic pro-inflammatory markers and cytokines in KC

**Figure 6:**
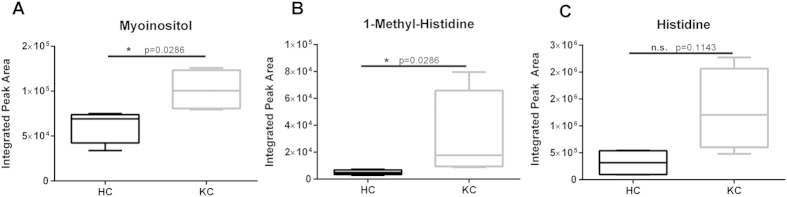
Pro-inflammatory biomarkers isolated in saliva from healthy controls (HC) and Keratoconus (KC) patients by LC/MS-MS. Myoinositol and histidine-derivatives have been associated with inflammation. (**A–C**) Myoinositol and 1-methyl-histidine salivary levels were significantly higher in KC patients with no significant variation in histidine. n = 4 for all data, with each n representing a different patient. The boxplot representation depicts the central rectangle spanning the interquartile range with a line showing the median. Whiskers above and below the central box represent the maximum and minimum data points, respectively. Statistical significance was determined using a Mann-Whitney two-tailed T-test with p ≤ 0.05 considered statistically significant.

Estrogens have been shown to directly modulate expression of pro-inflammatory cytokines, including IL-6 and TNF-α, within corneal epithelial cells^41^. Myoinositol and 1-methyl-histidine are metabolites that have been associated with pro-inflammatory responses *in vivo*^42^. Since estrone and DHEA-S levels were altered in our KC samples, we sought to determine if the differences in hormone levels contribute to a pro-inflammatory response. We measured significant increase of myoinositol (1.6-fold, p = 0.0286) and 1-methyl-histidine (6.2-fold, p = 0.0286) in KC samples with no difference in histidine levels (Fig. 6, p = 0.1143).

**Figure 7:**
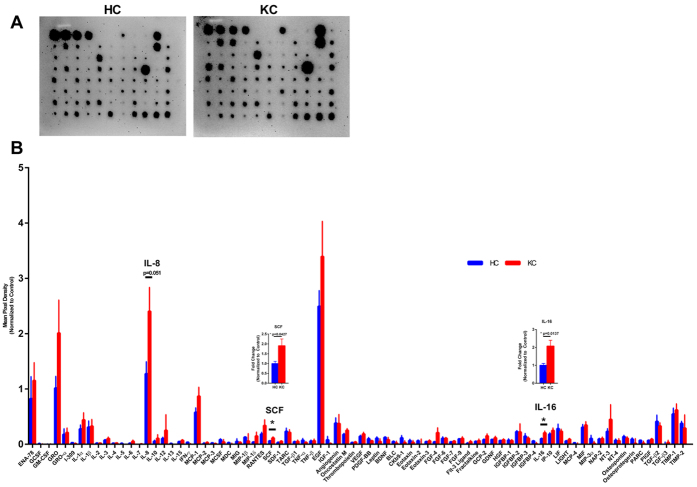
Relative amounts of 80 cytokines and growth factors present in human saliva isolated from healthy controls (HC) and Keratoconus (KC) patients measured using a microarray. (**A**) Representative microarray membranes. (**B**) Quantification of all cytokines and growth factors detected on the microarray. Data normalized to the positive control bands present on each membrane. Statistical significance was determined using the Holm-Sidak method in a T-test, with alpha = 5.00%. n = 7 for HC and n = 9 for KC. Y-axis was set to 0, which cut off negative values. Error bars represent standard error of the mean.

In order to determine if systemic changes in pro-inflammatory cytokines and growth factors occurred in KC, we utilized a microarray capable of probing for 80 proteins present in saliva. The highest abundance of cytokines and growth factors secreted in human saliva were epidermal growth factor (EGF), interleukin-8 (IL-8), GRO or chemokine (C-X-C motif) ligand 1 (CXCL1), and epithelial-derived neutrophil-activating peptide-78 (ENA-78) (Fig. 7). Interestingly, we measured significantly increased stem cell factor (SCF) (1.9-fold, p = 0.0427) and interleukin-16 (IL-16) (p = 2.1-fold, 0.0137) in KC saliva samples compared to healthy controls (Fig. 7B). SCF is an important growth factor that is critical for promoting differentiation of different cell types, including progenitor cells to myeloid and erythroid-derived cells^43^. SCF functions as a ligand for the c-Kit tyrosine kinase receptor (c-Kit), which is important in propagating pro-survival signals to induce proliferation and differentiation^43^,^44^. Our results suggest that SCF plays a role in KC and potentially provides a link between pro-inflammatory processes and KC pathobiology.

**Figure 8:**
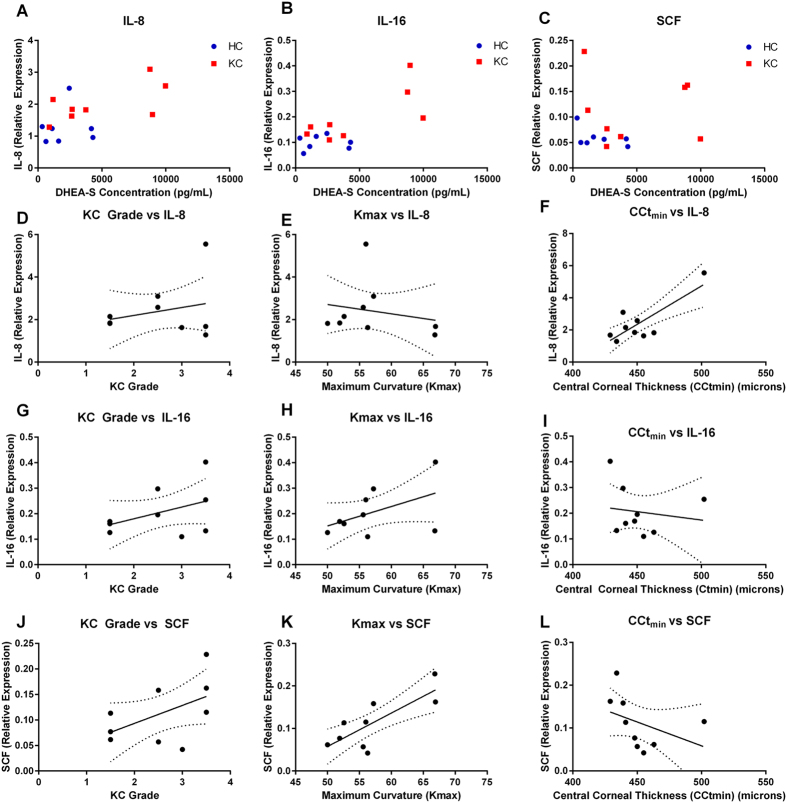
Pro-inflammatory cytokines and growth factors, IL-8, IL-16, and stem cell factor (SCF) correlated with hormone levels and KC severity. (**A**) IL-8, (**B**) IL-16, and (**C**) SCF expression plotted relative to DHEA-S levels. IL-8, IL-16, and SCF expression relative to (**D–F**) KC grade, (**G–I**) Maximum corneal curvature (Kmax), (**J–L**) Minimum central corneal thickness (CCt_min_) measured in microns. Best-fit linear line plotted with 90% confidence intervals (dotted lines). Expression of IL-8, IL-16, and SCF measured using densitometry from a microarray. n = 7 for HC and n = 9 for KC. Clinical features (Kmax, and CCt_min_) were not collected for all HC and therefore were excluded from (**D–L)**.

Furthermore, we analyzed these three elevated systemic pro-inflammatory molecules relative to KC severity, as measured by clinical standards. We quantified KC severity (KC grade) versus expression of IL-8, IL-16, and SCF. We found a slight linear response with increasing KC grade and IL-8 levels, but without any correlation with Kmax and an increase in corneal thickness with the highest IL-8 concentration (Fig. 8D–F). Elevated IL-16 levels correlated with more severe KC features with increased KC grade, maximum curvature, and a slight reduction in corneal thickness (Fig. 8G–I). IL-16 is a lymphotactic factor able to recruit activated T cells and other CD4-expressing cells. It appeared that increased SCF levels exhibited the highest linearity with severity of disease, with elevated SCF expression correlating with higher KC grade, maximum curvature, and thinner corneal stroma (Fig. 8J–L). This data suggests that a systemic inflammatory process, involving IL-16 and SCF, may contribute to increased KC severity and reveals a novel connection between elevated hormonal levels and inflammation in KC.

## Discussion

Regulation of hormone levels occurs throughout development, from birth to adolescence, puberty to adulthood, and then postmenopausal to the elderly^45^,^46^,^47^,^48^,^49^. These stages of development are correlated with significant variations in levels of DHEA-S and estrogens with climatic increases into puberty and gradual reductions with aging^7^,^50^,^51^. KC is a corneal-thinning disease that is primarily diagnosed from post-pubescence to adulthood, with our study showing an average age of 26 (±1.14) years old in males and 34 (±1.75) years old in females. It is known that KC progression often stabilizes at middle age^16^ with a younger onset of disease associated with a more severe disease condition requiring penetrating keratoplasty^52^. The factors that contribute to stabilization of the disease are unknown. Our results suggest that hormonal regulation may play a central role in the KC pathology. We found significant increases in salivary DHEA-S levels and reduced estrone levels in KC patients independent of gender. No apparent correlation between hormone concentration and increased severity of KC was detected, which led us to suspect that perhaps hormone levels modulate other systemic factors that may affect the stromal microenvironment, such as inflammatory factors and cellular metabolism, which might promote KC progression.

Elevated DHEA levels have been found to increase immune function by blocking endogenous glucocorticoid activity^53^,^54^. Our results showed elevated DHEA-S levels correlated with increased IL-16 and SCF, with higher IL-16 and SCF corresponding to increased KC severity. IL-16 has been found to be elevated along with other cytokines in the vitreous of pseudophakic eyes following cataract surgery^55^ and is known to play a complex functional role in the regulation of the immune response through interaction with T cells and dendritic cells^56^. SCF has a much different role in immune regulation as a ligand for the tyrosine receptor, c-kit, and is involved in promoting differentiation of lymphoid, myeloid, erythroid, and megakaryocytic cell lineages^57^. Upregulation of both IL-16 and SCF suggest an important role for the immune system and cell differentiation patterns in the KC pathobiology. Interestingly, we did not observe significant upregulation of other pro-inflammatory cytokines suggesting that IL-16 and SCF are specific factors that may contribute to KC severity. Systemic metabolic features of KC corresponded with the upregulation of myoinositol and 1-methyl-histidine as potential biomarkers in agreement with the presence of systemic pro-inflammatory factors.

KC severity is characterized by the clinical grade, maximum curvature, and minimum corneal thickness. Corneal thickness is a measure of extracellular matrix deposition in the cornea with gradual thinning of the stroma leading to KC development. We identified a direct link between elevated IL-16 and SCF and thinner KC corneas, increased curvature, and KC grade suggesting elevated inflammatory factors may contribute to disease severity. We have previously identified KC as a metabolically-driven disease in primary corneal stromal cells derived from KC patients with increased oxidative stress and reduced matrix thickness^35^,^58^. Our current study, for the first time to the authors’ knowledge, utilizes human saliva samples to correlate clinical findings with metabolic changes in KC. Our results suggest that KC is at least partially a systemic disease driven by altered hormones contributing to stromal thinning in the KC cornea. Further studies are clearly needed with the participation of larger number of KC patients. This work represents a significant step in assisting with KC prognosis and drug development.

## Methods

### Study Design

A cross-sectional, controlled laboratory experimental study was conducted from clinical data and saliva samples collected from KC patients and healthy controls. Given that KC has a prevalence of 1:2000^16^, our study attempted to isolate the maximum number of saliva samples from two centers (Aarhus University Hospital, Denmark and Dean McGee Eye Institute, USA). Inclusion/exclusion criteria for data collection were established at the onset of data analysis to exclude clinical data from patients who had previously received collagen crosslinking or undergone penetrating keratoplasty. Robust regression and Outlier removal (ROUT) was utilized to determine outliers post-hoc, as previously described^59^. Not all saliva samples yielded values within the standard curve and were therefore excluded from analysis. Replicates varied between experiments and are defined in the appropriate figure legends. Sample replicates were analyzed and screened individually with no pooling of samples.

### Ethics and Inclusion Criteria

The ethics committee of both the University of Oklahoma and the Central Denmark Region approved all experimental protocols reported in this study. IRB approvals (#3450 and #1-10-72-77-14) were obtained by both ethic committees and written informed consent was received from participants prior to inclusion in the study. Demographics data (age, gender, CCt_min_, Kmax, KC grade) was provided by a clinician with no identifiers linked to clinical information. This study met the tenets of the Declaration of Helsinki. Inclusion criteria for healthy controls required absence of KC diagnosis or other corneal diseases. Inclusion criteria for KC patients required diagnosis of KC by a certified ophthalmologist and absence of other ophthalmic conditions. Healthy controls and KC patients were age-matched as closely as possible with average ages of 35 ± 3.138 and 29 ± 1.064 years, respectively (p = 0.0896, Supplemental Fig. S7A). Kmax and CCt_min_ values for controls were only available for n = 8 patients and are reported in Supplemental Fig. S7B,C.

### Saliva Collection

Patients were requested to rinse mouth with water prior to sample collection. Saliva samples (~2mL) were collected in plastic tubes by passive droll and frozen immediately^60^. Prior to processing, saliva samples were aliquoted and stored at <80 °C until further use.

### Salivary ELISAs

The following commercial immunoassay kits were used to detect hormone levels present in saliva: salivary estrone enzyme immunoassay kit (1–3202), salivary estriol/HS estriol enzyme immunoassay kit (1–1802), high sensitivity salivary 17β-estradiol enzyme immunoassay kit (1–3702), salivary DHEA-S enzyme immunoassay kit (1–1252) (Salimetrics, State College, PA). Briefly, saliva samples were maintained on ice and centrifuged at 3000 rpm (4 °C) for 15 minutes to pellet debris and mucins. 100 μL of neat saliva, standard, or control and the re-constituted conjugate was added to the appropriate well and incubated for 1 hour at room temperature or overnight at 4 °C with rocking. Wells were then washed 4× with provided wash buffer and incubated with the TMB solution for 30 minutes covered and at room temperature. The stop solution was then added, mixed briefly, and read in a plate reader at 450 nm. A 4-parameter non-linear regression curve fit was used to calculate the concentrations of controls and unknowns extrapolated from the standard curve.

### Salivary Metabolite Isolation

Metabolites were isolated as previously described^35^. Collected saliva samples were centrifuged at 3000 rpm (4 °C) for 15 minutes to pellet debris and mucins. 500 μL of the supernatant were added to 4 mL of cold 80% methanol and incubated on dry ice for 15 minutes. Samples were then centrifuged and metabolites isolated (repeated 3×), dried using a speedVac, and stored at −80 °C until further use.

### Mass Spectrometry Processing

Isolated metabolites were dissolved in 5 μL of HPLC-pure water and injected into a hybrid 5500 QTRAP triple quadrupole mass spectrometer (AB/SCIEX) coupled to a Prominence UFLC system (Shimadzu) using an Amide HILIC column (Waters) and analyzed with selected reaction monitoring (SRM) with positive/negative polarity switching. Peak areas from the total ion current were quantified for 297 metabolites^39^. Only metabolites that were expressed in n ≥ 3 samples were considered for further analysis. CIMminer software available from the genomics and bioinformatics group (http://discover.nci.nih.gov/cimminer/home.do) was used to generate the heat map following input of raw net intensity data for all metabolites with n ≥ 3 samples and clustering according to abundance.

### Human Cytokine Antibody Array

Manufacturer’s protocol was followed for the cytokine microarray (ab133998, Abcam, Cambridge, MA). Briefly, saliva sample was centrifuged at 3000 rpm (4 °C) for 15 minutes to pellet debris and mucins. Following blocking of the membrane, 1 mL of supernatant (neat) was incubated with the membrane overnight at 4 °C with rocking. Following washing, reconstituted 1× Biotin-Conjugated Anti-Cytokines was incubated with membrane for 2 hours at room temperature followed by washing. 1× HRP-Conjugated Streptavidin was then incubated with membrane for 2 hours at room temperature. Detection buffer C and D were added to the washed membrane and immediately imaged. Densitometry measurements were performed and background intensity was subtracted from the raw net intensity. In order to compare values between microarrays, all values were normalized to the average intensity of the positive control wells (1–4) on the same membrane. Values for each probe were then averaged and plotted.

### Statistical Analysis

Statistical analysis was performed using Graph Pad Prism 6 and a Mann-Whitney unpaired T-test or Holm-Sidak method in a T-test were used to determine significance. A p-value < 0.05 was considered statistically significant.

## Additional Information

**How to cite this article**: McKay, T. B. *et al.* Endocrine and Metabolic Pathways Linked to Keratoconus: Implications for the Role of Hormones in the Stromal Microenvironment. *Sci. Rep.* **6**, 25534; doi: 10.1038/srep25534 (2016).

## Supplementary Material

Supplementary Information
