# Supplementary material for: Endocrine and Metabolic Pathways Linked to Keratoconus: Implications for the Role of Hormones in the Stromal Microenvironment
Source: Sci Rep. 2016 May 9;6:25534. doi: 10.1038/srep25534 (PMC4860577; doi:10.1038/srep25534)
Supplement: Supplementary Information [file srep25534-s1.doc]

**Endocrine and Metabolic Pathways Linked to Keratoconus: Implications for the Role of Hormones in the Stromal Microenvironment**

*Tina B McKay^1^, Jesper Hjortdal^2^, Henrik Sejersen^2^ John Asara^3^, Jennifer Wu^4^, Dimitrios Karamichos^1,4,*^*

*^1^Department of Cell Biology/ University of Oklahoma Health Sciences Center,*

*^2^Department of Ophthalmology, Aarhus University Hospital, Aarhus C DK-800, Denmark,* ^3^*Division of Signal Transduction, Beth Israel Deaconess Medical Center, Boston, Massachusetts, USA,*

*^4^Department of Ophthalmology/ Dean McGee Eye Institute, Oklahoma City, OK 73104, USA.*

**^*^ Corresponding author:** Dimitrios Karamichos, Ph.D., Department of Ophthalmology/Dean McGee Eye Institute, University of Oklahoma Health Sciences Center, Oklahoma City, OK 73104, USA. Tel.: +1 405 271 4019; fax: +1 405 271 8128; e-mail: [dimitrios-karamichos@ouhsc.edu](mailto:dimitrios-karamichos@ouhsc.edu)

**Supplemental Figures**

**
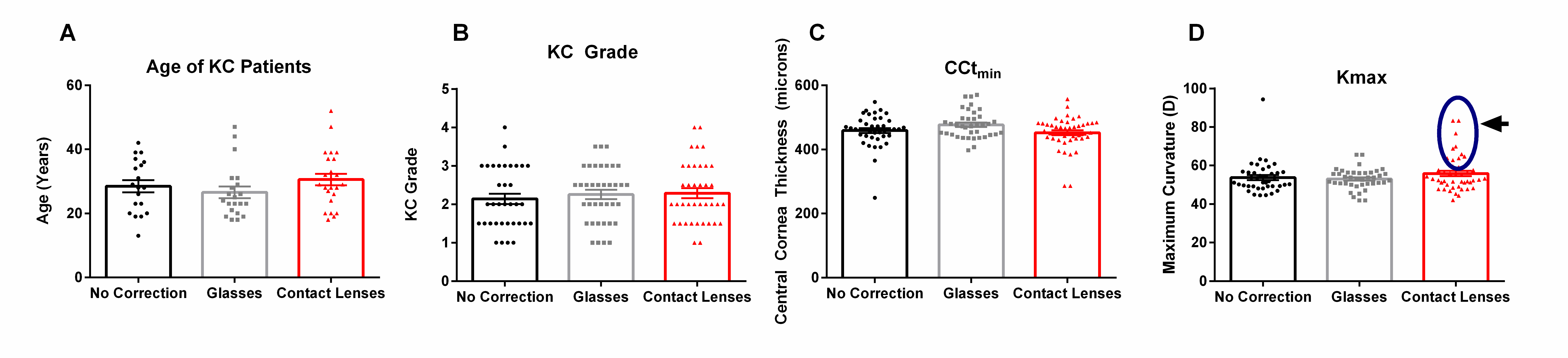
**

**E**

**Figure S1. Demographics of the Keratoconus (KC) population based on treatment for visual acuity.**

(A) Treatment plotted versus age in years, (B) Severity of KC (KC grade), (C) Minimum central corneal thickness (CCt_min_) measured in microns, and (D) Maximum corneal curvature (Kmax). Blue circle and arrow show KC patients in the contact lens subgroup with increased corneal curvature (Kmax).

**
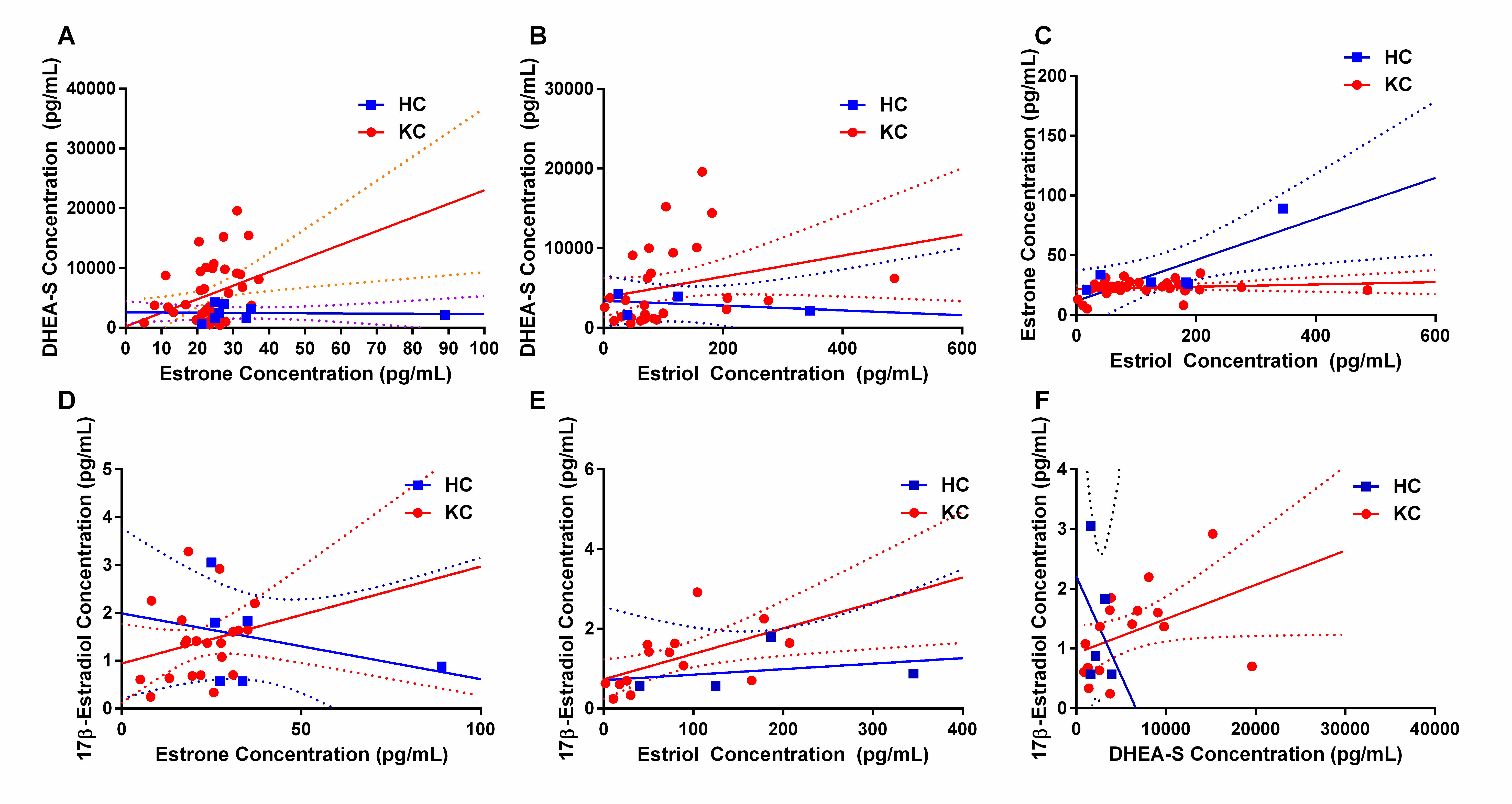
**

**Figure S2. Correlational plots of hormone levels showing variability between healthy controls (HC) and Keratoconus (KC).**

(A) Estrone versus dehydroepiandrosterone (DHEA-S) levels, (B) estriol versus DHEA-S levels, (C) estriol versus estrone levels, (D) estrone versus 17β-estradiol levels, (E) estriol versus 17β-estradiol levels, and (F) DHEA-S versus 17β-estradiol levels. KC patients showed the highest DHEA-S levels compared to HCs. Each dot represents a separate patient (HC in blue and KC in red). The best-fit line was plotted and 90% confidence intervals shown with dotted lines.


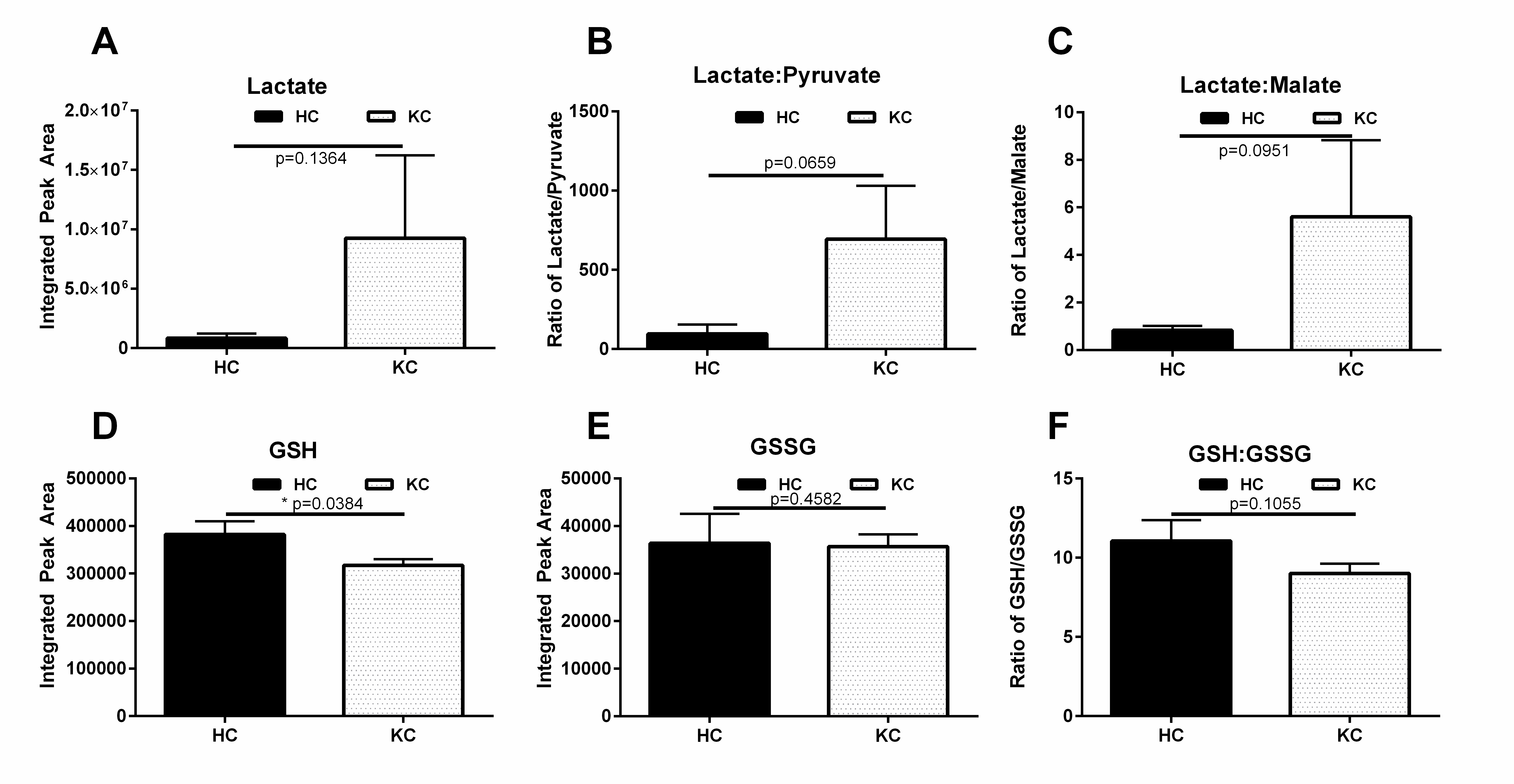


**Figure S3. Metabolic profile of saliva isolated from healthy control (HC) and Keratoconus (KC) patients measured by LC/MS-MS.**

(A) Lactate levels were slightly elevated in KC samples compared to HC suggesting systemic alterations in energy production. In order to determine lactate production relative to citric acid cycle conversion, we plotted ratios of (B) lactate to pyruvate and (C) lactate to malate and found increased ratios in both suggesting reduced shuttling of pyruvate into the citric acid cycle and then to oxidative phosphorylation, but rather conversion to the anaerobic product, lactate. (D) Measure of reduced glutathione (GSH) and (E) oxidized glutathione (GSSG) levels in saliva samples from HC and KC patients. (F) The ratio of reduced to oxidized glutathione (GSH:GSSG) is a measure of oxidative stress. We see a slight reduction in both GSH and GSH:GSSG suggesting that KC patients may have elevated systemic oxidative stress. n=4, with each n representing a different patient donor. Error bars depict standard error of the mean. Statistical significance was determined using an unpaired one-tailed T-test, with p<0.05 considered statistically significant.


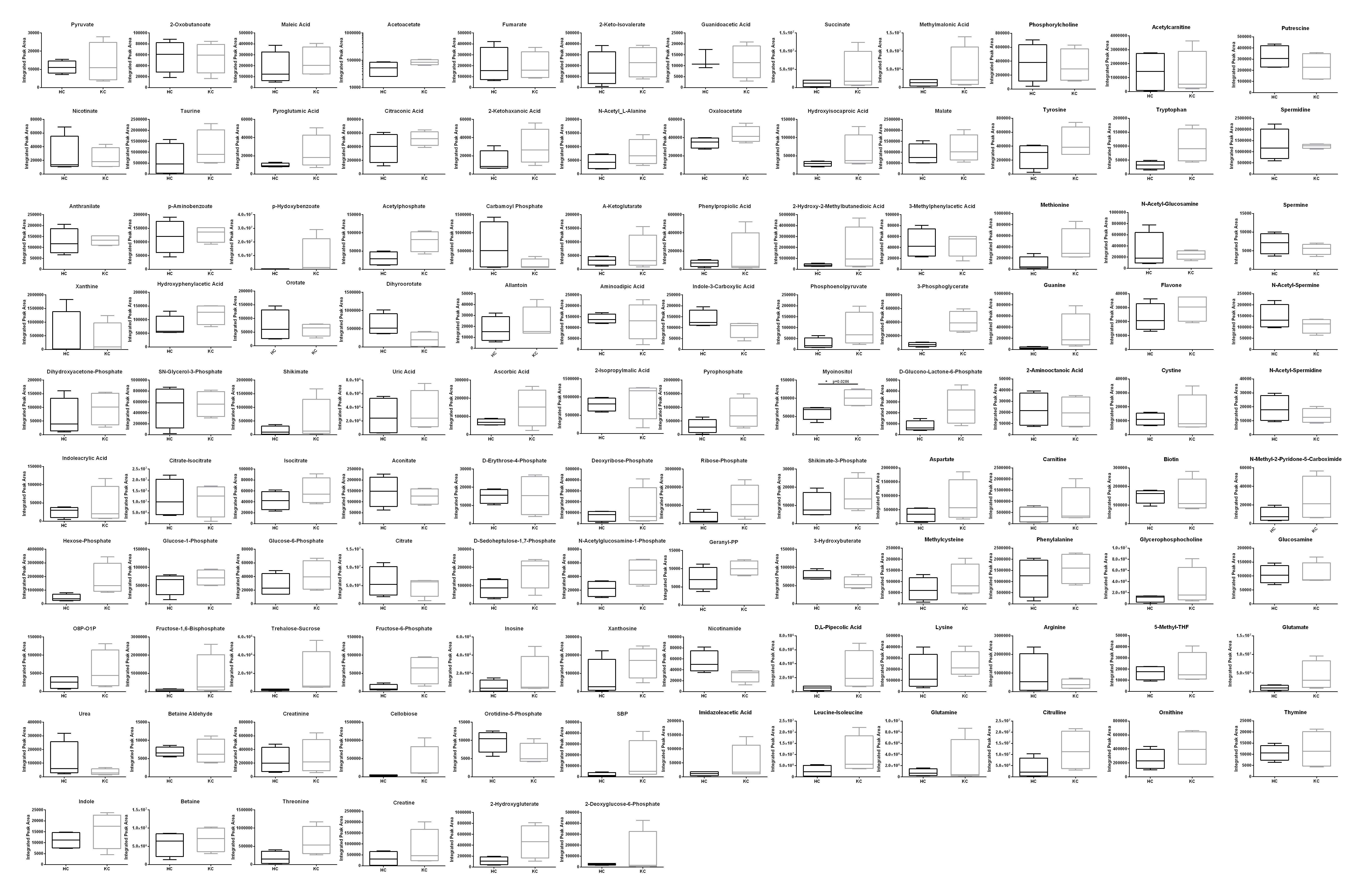


**Figure S4. 114 Metabolites identified by LC/MS-MS in saliva samples isolated from healthy controls (HC) and Keratoconus (KC) patients.**

n>3, with each n representing a different patient donor. p<0.05 was considered statistically significant.


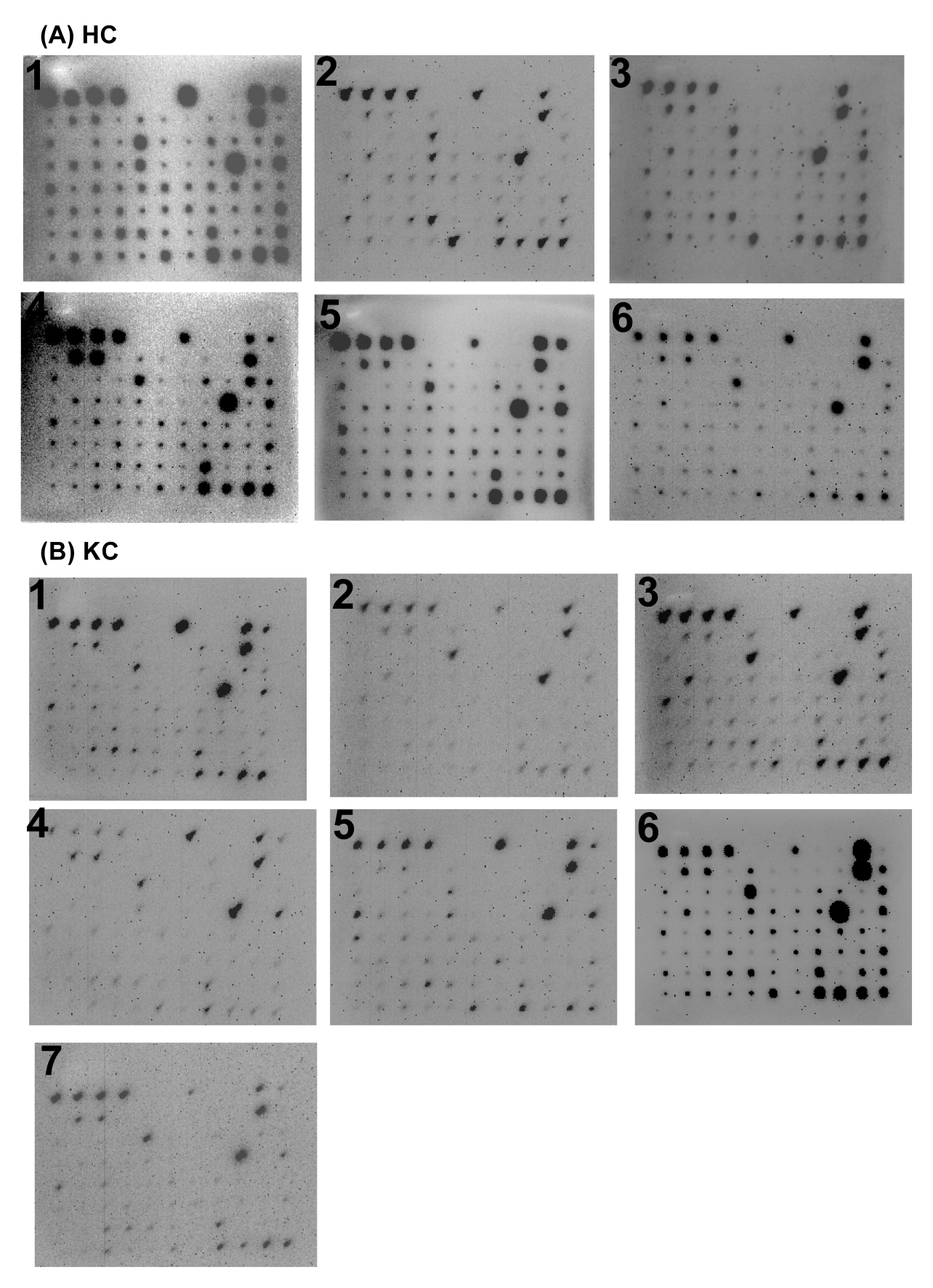


**Figure S5. Representative cytokine microarrays probed with human saliva isolated from (A) healthy controls (HC) and (B) Keratoconus (KC) patients.**

**
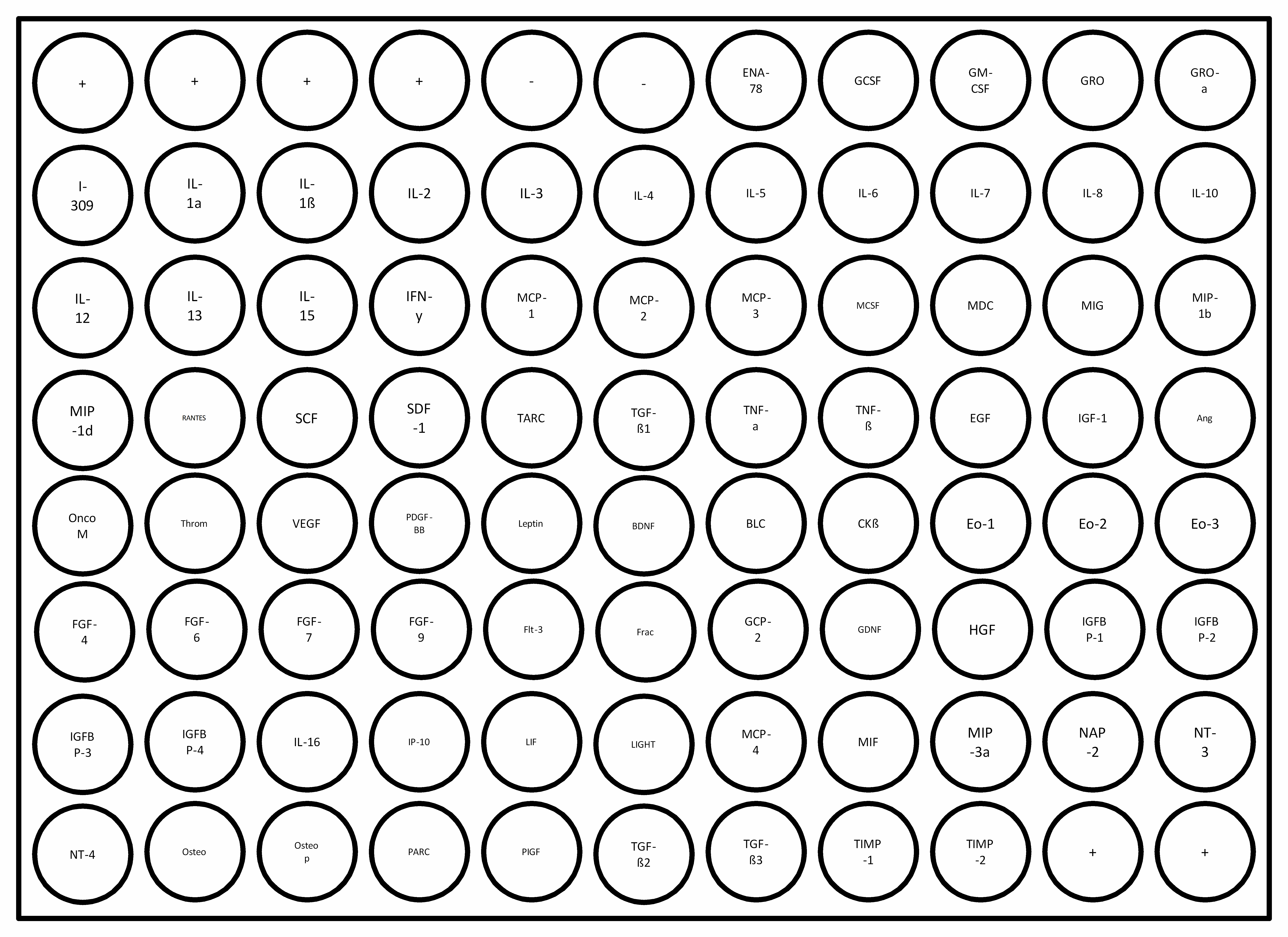
**

**Figure S6. Key to cytokine microarray showing location of each probe on the membrane.**


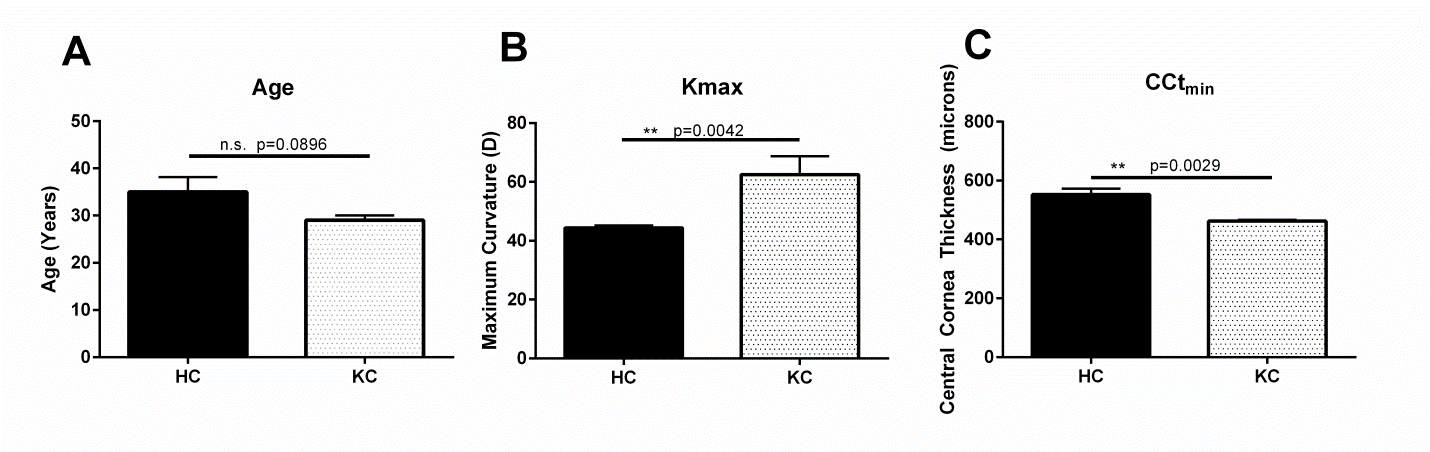


**Figure S7. Average age, Kmax, and minimum central corneal thickness (CCt_min_) in healthy controls (HC) versus KC patients.**

The average age of HC (35+3.138 years) compared to KC (29+1.064 years) (n=14 for HC, and n=64 for KC). (B) Kmax and (C) CCt_min_ were not obtained for all HCs with n=8.
